# Surgical and Oncological Outcome of Laparoscopic Resection of Colorectal Cancers: A Single-Center Experience

**DOI:** 10.7759/cureus.58849

**Published:** 2024-04-23

**Authors:** Sarhang H Muhammed, Neyan M Asad, Azhy M Dewana, Baderkhan S Ahmed, Ali Al-Dabbagh

**Affiliations:** 1 General Surgery, Hawler Medical University, College of Medicine, Erbil, IRQ; 2 General Surgery, Rizgary Teaching Hospital, Erbil, IRQ; 3 General Surgery, Zheen International Hospital, Erbil, IRQ

**Keywords:** kurdistan region of iraq, surgical outcome, oncological outcome, colorectal cancer, laparoscopy

## Abstract

Background: Laparoscopy is one of the major advances in surgery in the last 30 years and has many benefits. Although laparoscopy was initially used for resection of benign colon lesions, it is now widely used for colorectal cancer resections after strong evidence has confirmed its safety and efficacy. We aim to report both the surgical and oncological outcomes of our first series of laparoscopic colorectal cancer resections.

Methods: In 2013, a laparoscopic colorectal resection service was established in northern Iraq at Zheen Hospital, Erbil. Data from all consecutive colorectal cancers were collected. Patients with locally advanced diseases and those who required emergency operations for bowel obstruction or perforation were excluded. We analyzed demographic, operative, postoperative, and histopathological data for all patients who were included in the study.

Results: A total of 124 patients with colorectal cancers presented to our unit between January 2013 and January 2023. Only 112 patients fulfilled the inclusion criteria and underwent laparoscopic resections. The median age of the patients was 54.5 years. The majority of patients were men (n=62; 55.4%). In 39 patients (35%), the cancer was located in the sigmoid; in 33 patients (29.5%) the cancer was in the rectum. Laparoscopic anterior resection was the most common procedure (n=50; 45%), followed by right hemicolectomy in 17 cases (15.1%). The conversion rate to open surgery was 8% (nine cases). The most common causes of conversion to open surgery were dilated bowel loops and tumour adherence to other structures. The mean operative time was 190 minutes and the mean hospital stay was three days. No complications were reported in 94 patients (84%). Among the complications, wound infection was seen in seven patients (7.8%). There were six anastomotic leaks (6.7%). The mean number of lymph nodes harvested was 13. In 70 patients (62.5%), the lymph node count was ≥12 with a median of 13. The mean distal resection margin was 6 cm and 2.5 cm for colon and rectal resections, respectively.

Conclusion: This study reveals that laparoscopic resection for colorectal cancers is surgically practicable and safe with the benefits of a short hospital stay, adequate resection margins, and adequate lymph node yield.

## Introduction

Laparoscopic colon resection has become the mainstay treatment option for colorectal cancer in the developed world. It has been well-recognized as the first treatment option for colorectal cancer by most surgical associations. The benefits of laparoscopy include a lower incidence of postoperative ileus, less postoperative pain and a reduction in the need for analgesics, shorter period of diet restrictions, shortened hospital stay, earlier return to daily activity, less wound-related morbidity, improved cosmetic results and a possible reduction in adhesion formation [1]. The first colorectal laparoscopic resection was reported by Jacobs et al. in 1991 [2]. Watanabe et al. were the first to report the results of laparoscopic colorectal resection for colon cancer in 1993 [3]. Since then, multi-centric randomized trials have shown that laparoscopy is a safer procedure for colorectal cancer resection than open surgery in terms of five-year overall and disease-free survival rates [4-6]. Further evidence strongly suggests that there are no statistically significant differences between open and laparoscopic surgeries regarding the incidence of tumour local recurrence, distant metastases, or disease-free survival [7-10]. Currently, laparoscopic resection for even large tumours is recognized to be as effective as open resection [11,12]. These findings led to wide acceptance of laparoscopy even for larger and advanced tumours. There is an obvious trend toward laparoscopy, although at a slow pace. In Great Britain, 40% of colorectal resections were performed laparoscopically in 2012 compared to only 5% in 2005 [13]. Ten years later, the rate increased to 74% [14].

A modern surgical unit for colorectal diseases was established in 2012 at Zheen Hospital in Erbil, northern Iraq. The unit is the first of its type in Iraq; it is well-equipped and run by a trained team of colorectal surgeons. We started laparoscopic colorectal resection services for both malignant and benign conditions in 2013; since then, a total of 210 laparoscopic resections have been performed. The initial draft of this article was previously posted to the ResearchSquare preprint server on September 20, 2023 [15].

The objective of this study is to analyze the results of the laparoscopic surgical interventions that have been performed on patients with colorectal cancers at our colorectal unit.

## Materials and methods

In this prospective analysis, all consecutive patients with malignant colorectal tumours who presented to our unit (including referrals) and underwent laparoscopic colorectal resection from January 2013 to December 2022 were included in this study. Patient’s hospital records were reviewed to retrieve the demographic and clinical data including preoperative records, cancer staging, intraoperative and procedure details and postoperative surgical, histopathological and subsequent clinical follow-up data.

During the period of the study, 124 patients presented with colorectal cancer to our unit, of those, 12 patients were deemed unsuitable for laparoscopic resection after preoperative assessment. Accordingly, they were planned for open surgery and were excluded from the study. Laparoscopic intervention for colorectal cancers was not performed for the following: emergency cases (for example, large bowel obstruction by cancer or colon perforation and peritonitis), locally advanced T4 tumours confirmed by preoperative CT scan requiring extra anatomical dissection, recurrent cancer, and presence of medical contraindications to laparoscopy.

Preoperative workup

All patients underwent colonoscopy and tissue histology. A staging CT scan of the chest, abdomen, and pelvis was performed to detect any distant spread. For local staging of rectal cancer, a pelvic MRI was performed. Tumours were localized using both colonoscopy and CT scan. The procedure and possibility of a stoma were fully explained to all patients, and informed consent was obtained.

All patients were given 4,000 or 6,000 IU enoxaparin sodium subcutaneously on the evening before surgery, which continued for five days after the operation. No mechanical bowel preparation was used for any patient except for a single phosphate rectal enema 30 minutes before surgery in cases of rectal cancer. The rectal tumour was defined by location within 15 cm from the anal verge.

Patients were discussed in gastrointestinal multidisciplinary (GIT-MDT) meetings. All rectal cancer patients with tumours below 12 cm from the anal verge were referred for neoadjuvant treatment. Because of the inadequate and irregular supply of neoadjuvant medications, not all patients with rectal cancer had the chance to receive this treatment and were planned for surgery without neoadjuvant treatment instead.

Procedure

At the induction of anaesthesia, a single dose of 1 gm of ceftriaxone and 500 mg of metronidazole were administered and later continued for one week postoperatively. All patients wore thromboembolic prevention stockings and underwent intermittent pneumatic compression of the calf during surgery. Two ports were placed lateral to the umbilicus and suprapubic positions. Pneumoperitoneum was achieved with direct vision port insertion, an extra port in the right lower quadrant or left lower quadrant in left-sided resection and left-sided resections respectively. Left and right colon segments were mobilised by medial to lateral dissection using Ligasure® (Covidien Inc., Dublin, Ireland).

The procedure routinely involved a proximal division of vessels, the inferior mesenteric artery for the left colon and rectum, and the ileocolic, right colic, and right branch of the middle colic artery for the right colon. The right hemicolectomy specimen was delivered through a 5 cm transverse incision to the right side of the umbilicus while a 5 cm suprapubic incision was used to deliver left-sided and total colectomy specimens. Continuity was restored by an extra-corporeal, stapled, functional, side-to-side anastomosis using linear stapling devices. Colon stump perfusion was assessed by visual inspection before fashioning the anastomosis. The splenic flexure was mobilized when deemed necessary. Reconstruction was performed with a circular stapling device to perform a colorectal anastomosis after anterior resections or ileorectal anastomosis following total colectomy.

For mid and lower rectal lesions, total mesorectal excision (TME) was performed. In cases of low anterior resection when the anastomosis was within 2 cm from anorectal junction, a protective diversion loop ileostomy was performed. All colorectal anastomoses were tested for a leak by underwater air insufflation. None of the patients had a nasogastric tube. A pelvic drain was used only after rectal surgery and removed anytime within 24 hours.

Conversion was defined as stopping the laparoscopy and continuing the dissection via a laparotomy wound larger than the incision used to extract the specimen. Conversion to open surgery was required in case of difficulty proceeding with laparoscopy in the presence of dilated bowel loops, T4 tumours attached to other organs requiring extra-anatomical dissection that was not shown on preoperative CT scan, or patient characteristics such as a thick mesentery and omentum in cases of obesity.

Postoperative status and follow-up

All patients started an oral soft and liquid diet and mobilization from the first postoperative day. Urinary catheters and pelvic drains were removed on the first postoperative day unless indicated otherwise. After discharge from the hospital, patients were seen on postoperative Days 5 and 10 to record progress and the presence of morbidity. All patients were discussed in the GIT-MDT meetings to decide on adjuvant chemotherapy, and those who fulfilled the criteria were referred to the oncology unit. Patients thereafter were scheduled for regular biannual follow-ups with an ultrasound scan, CT scan of the chest, abdomen, and pelvis, and by measuring the carcinoembryonic antigen (CEA) level. Local recurrence was defined as any recurrence diagnosed or suspected at the site of the surgery or abdominal wound. Distant metastasis was defined when a new lesion appeared in the liver, lung, brain, or bone during follow-up assessments. Early death was defined as death within 30 days post-operation.

Statistical analysis

Data were presented in numbers and proportions. Mean ± standard deviation (SD) was calculated for the regularly distributed data, while median (range) was used for the non-regularly distributed data. Continuous data were compared using the independent sample t-test. A p-value of <0.05 was considered significant. Microsoft® Excel 2020 was used for the analysis. The study was approved by the Ethics Committee of the College of Medicine, Hawler Medical University, Erbil, Iraq.

## Results

A total of 112 patients with colorectal cancer were enrolled in this study; 62 (55.4%) were male and 50 (44.6%) were female. Their age ranged from 19 to 92 years with a median age of 54.5 years.

According to the American Society of Anesthesiologists (ASA) grade of health status, 56 (50%) patients had ASA I grade and only 10 patients (9%) had ASA III. The primary tumour was in the colon in 79 patients, while the remaining 33 patients had their primary tumours in the rectum. Neoadjuvant therapy was given to three patients with colon cancer and 11 patients with rectal cancer.

Concerning the operative details, anterior resection was the most frequently performed procedure (n=50; 45%) on lower sigmoid, rectosigmoid, and rectal lesions. The detailed clinical characteristics and intraoperative details of the enrolled patients are illustrated in Table 1. Regarding the operative time, the median duration of the laparoscopic interventions was 190 minutes, ranging from 130 to 280 minutes. After splitting the nine-year duration of this study into three sections, there was a significant difference (p=0.034) between the operation time of the first three-year period (mean 230 minutes) and the subsequent two periods (mean 190 minutes) (Table 2).

**Table 1:** Patients’ clinical characteristics and operative details Data is represented as n (%). ASA: American Society of Anesthesiologists

Characteristics	n (%)
ASA Score	
I	56 (50)
II	46 (41)
III	10 (9)
Tumor locations	
Right colon	21 (18.7)
Left colon	19 (17)
Sigmoid	26 (23.2)
Rectosigmoid	13 (11.6)
Rectum	33 (29.5)
Neoadjuvant therapy in rectal cancer	
Short course	5 (4.4)
Long course	6 (5.3)
Neoadjuvant therapy in colon cancer	3 (2.6)
Type of surgical resection	
Right hemicolectomy	17 (15)
Left hemicolectomy	14 (12.5)
Total colectomy	12 (10.7)
Anterior resection	50 (45)
Sigmoid colectomy	9 (8)
Abdominoperineal resection	8 (7)
Hartmann’s procedure	2 (1.8)

**Table 2:** Length of surgical interventions, hospital stay, and operative details SD: standard deviation, min: minutes

Variables	Median (Range)	Mean (±SD)	p-value
Duration of surgery, overall (min.)	190 (130-280)	N/A	N/A
Duration of surgery, first 3 years (min.)	N/A	230 ±35.53	0.034
Duration of surgery, last 6 years (min.)	N/A	190 ±30.27	
Hospitalization, all cases (days)	N/A	3 ±1	N/A
Hospitalization of complicated cases (days)	N/A	4 ±1.02	<0.001
Hospitalization of non-complicated cases (days)	N/A	2.9 ±1	
Number of harvested lymph nodes, all cases	13 (33-66)	N/A	N/A
Number of harvested lymph nodes, rectal tumours	13 (3-62)	N/A	N/A
Distal resection margin, colon tumours	6 (1-32)	N/A	N/A
Distal resection margin, rectal tumours	2.5 (0.5-5)	N/A	N/A

Patients with proper rectal cancers (n=33) were operated by TME for which anterior resection was performed in 25 patients (75.75%), abdominoperineal resection (APR) in seven patients (21.25%), and Hartmann’s procedure in one patient (3%). Among all laparoscopic interventions, nine (8%) were converted to open procedures either because of a locally invading tumour or dilated bowel loops in six and three patients, respectively.

In regard to postoperative events, the mean patients’ hospital stay was three days, ranging from two to seven days. Early death was encountered in one (0.9%) patient because of myocardial infarction. Postoperative morbidity was encountered in 18 (16%) patients. Six patients had a clinical anastomotic leak, of which four occurred following rectal cancer resection and were treated conservatively as they had a diversion ileostomy during the initial operation. The two other leaks occurred following colon resection, both of which required readmission and reoperation. The mean length of hospital stay in patients with complications was significantly higher than that in those with no complications (4 versus 2.9 days, respectively; p<0.001). Table 3 shows the postoperative events and summary of histopathological results of the resected cancers.

**Table 3:** Postoperative surgical and pathological outcomes Data is represented as n (%).

Postoperative and pathological data	n (%)
Complications	18 (16)
Paralytic ileus	4 (3.6)
Bleeding	1 (0.9)
Cardiovascular	1 (0.9)
Wound infection	7 (6.3)
Leak	6 (5.4)
Fistula	1 (0.9)
Reoperation rate	2 (1.8)
Histopathology results	
Well-moderately differentiated	97 (85.6)
Poorly differentiated and mucinous	13 (11.6)
No residual tumour (neoadjuvant therapy)	2 (1.8)
Final postoperative tumour stage	
Stage I	22 (20)
Stage II	41 (37.3)
Stage III	33 (30)
Stage IV	14 (12.7)

Tumour histopathology showed that 97 (85.6%) resected tumours were well-moderately differentiated adenocarcinomas. Two patients (1.8%) had complete resolution of the rectal tumour after receiving neoadjuvant chemoradiotherapy. The median number of lymph nodes harvested from colon and rectal specimens was 13 (range: 3-66); the median distal resection margin in colon resections was 6 cm, (range: 1-32 cm). The median number of lymph nodes harvested from the 33 rectal tumors was 13 (range: 3-62 cm), ≥12 lymph nodes were harvested in 70 patients (62.5%). The median distal resection margin in rectal masses was 2.5 cm (range: 0.5-5 cm) and was more than 2 cm in 20 (74%) patients (Table 2).

The mean period of the patient’s follow-up was 28 months, ranging from 2 to 103 months. During the follow-up, eight patients within the enrolled group were lost to follow-up. Recurrence of the tumour, either locally or distally, was recorded in 15 patients and death was recorded in 10 (8.9%) patients. However, the cause of death in seven patients was directly related to the cancer, while three patients died due to causes other than their cancer.

## Discussion

The current study reports the experience of one surgical unit for colorectal diseases in a developing country. The laparoscopic colon resection service was initiated after training a dedicated team and meeting the requirements. This minimally invasive technique is now widely adopted worldwide. Its efficacy and benefits have been shown in the literature with high-level evidence. What refuted the concerns that laparoscopy may jeopardize oncological outcomes were the conclusions of four major randomized controlled trials (COST, COLOR, Barcelona and MRC CLASSICC), which demonstrated that the surgical and survival outcomes were comparable to those of the open method [4,16-18].

In the current study, the mean age of patients at the time of a colorectal cancer diagnosis was 54.5 years, which is relatively lower than that in many other series [4,11-18]. This in turn explains the fact that 50% of our patients were ASA I and 41% were ASA II. It is well observed that less than 10% of rectal cancer patients had neoadjuvant treatment, which is very low compared to the standard management of rectal cancer. This is attributed to the shortage of these medications in our country, which cannot accommodate all referred cases.

Of all laparoscopic procedures, only 8% were converted to and completed by an open procedure. This is comparable with literature reports and even lower than some major studies reporting 21%, 25%, and 17% conversion rates [4,13-18]. These conversions were not influenced by the learning curve because they were spread throughout the period of the study. It is well known that laparoscopic colon cancer resection has a long learning curve that varies between surgeons and is affected by patient and operative complexity [19]. There was a significant difference between the operative times of the first three years of the study and each of the following two three-year periods.

According to the literature, the incidences of complications after laparoscopy are not significantly different from those after open surgery [20,21]. Overall, in the current cohort, early postoperative morbidity was reported in 16% of patients. Of the six patients who had anastomotic leaks, only two patients required readmission and reoperation. The other four patients had a long course of neoadjuvant chemoradiotherapy and had a de-functioning ileostomy at initial resection and were managed conservatively. The other complications were mild and were treated conservatively. Early death was recorded in one (0.9%) patient who was ASA III and had an acute myocardial infarction on Day 1 postoperatively. These findings partly coincide with other reported morbidities ranging from 7-30% [13,22-24]. Some earlier studies which included a larger number of patients reported even higher morbidity rates reaching 60-80% [4,17,18].

The mean length of hospital stay of four days compares favourably with other series. This is lower than the ACPGBI figure of 10 days for open elective colon resection [25]. Some studies reported 5 and 6.6 days [24,26]. The longer hospital stays in some reports may be attributed to an ageing population and the presence of comorbidities. We tended to discharge patients as soon as possible according to our enhanced recovery after surgery (ERAS) protocol and schedule two or three postoperative outpatient reviews within 10 days after the operation. Morbidity had a negative influence on the length of hospital stay. Patients stayed significantly longer when they had complications.

Postoperative surgical and pathological outcomes are acceptable in the form of clear resection margins and nodal yield. The distal resection margin in colon resections was <5 cm in 21.8% of cases and <2 cm in 25.5% of rectal resections. Only in one rectal resection, the distal resection margin was <1 cm. The mean lymph node yield of 13 is more than the recommended 12 lymph nodes [27]. In 37.5% of cases, the lymph node count was <12. The lowest nodal yield was three lymph nodes. Such low counts were observed in patients who had resections of recurrent tumours or in those who received neoadjuvant chemoradiotherapy [27]. Moreover, our pathologists harvest lymph nodes from the resected tumours by palpation method only, without using any fat-clearing methods to maximize nodal yield, as a result, even small nodes can be missed [28]. Unfortunately, circumferential resection margins are not routinely performed by every pathologist. This is why we lack some data. Only one patient with poorly differentiated mucinous adenocarcinoma had surgical wound recurrence, and two patients had pelvic recurrences. No anastomotic recurrence was reported in this series.

In the current cohort, eight patients were lost to follow-up; this is because we receive patients from distant geographical locations which makes it more difficult for some patients to attend regular follow-ups. Moreover, we acknowledge that this series is limited in some aspects, most importantly, the relatively small number of patients and the absence of resources to meet international guidelines regarding neoadjuvant treatment and histopathology resources. However, this study shows acceptable surgical and oncological outcomes while supporting the benefits of laparoscopy.

## Conclusions

In conclusion, the initiation of laparoscopic colorectal resection for cancer is feasible with acceptable outcomes even in countries with limited resources. The study showed comparable low complications, short hospital stays, adequate resection margins, and adequate lymph node yields. These results support the fact that laparoscopic colon resection for cancer can be safely completed, providing the benefits of laparoscopy without compromising surgical and oncological outcomes.
